# Boltzmann’s Theorem Revisited: Inaccurate Time-to-Action Clocks in Affective Disorders

**DOI:** 10.2174/1570159X22666240315100326

**Published:** 2024-03-18

**Authors:** Sari Goldstein Ferber, Aron Weller, Hermona Soreq

**Affiliations:** 1 Psychology Department and The Gonda Brain Research Center, Bar-Ilan University, Ramat Gan, Israel;; 2 Department of Psychological and Brain Sciences, University of Delaware, Newark, DE, USA;; 3 The Edmond & Lily Safra Center for Brain Sciences, The Hebrew University of Jerusalem, Jerusalem, Israel;; 4 The Alexander Silberman Institute of Life Sciences, The Hebrew University of Jerusalem, Jerusalem, Israel

**Keywords:** Biological clocks, brain timescales, goal-oriented behavior, psychopathology, logic gates, boltzmann’s theorem

## Abstract

Timely goal-oriented behavior is essential for survival and is shaped by experience. In this paper, a multileveled approach was employed, ranging from the polymorphic level through thermodynamic molecular, cellular, intracellular, extracellular, non-neuronal organelles and electrophysiological waves, attesting for signal variability. By adopting Boltzmann’s theorem as a thermodynamic conceptualization of brain work, we found deviations from excitation-inhibition balance and wave decoupling, leading to wider signal variability in affective disorders compared to healthy individuals. Recent evidence shows that the overriding on-off design of clock genes paces the accuracy of the multilevel parallel sequencing clocks and that the accuracy of the time-to-action is more crucial for healthy behavioral reactions than their rapidity or delays. In affective disorders, the multilevel clocks run free and lack accuracy of responsivity to environmentally triggered time-to-action as the clock genes are not able to rescue mitochondria organelles from oxidative stress to produce environmentally-triggered energy that is required for the accurate time-to-action and maintenance of the thermodynamic equilibrium. This maintenance, in turn, is dependent on clock gene transcription of electron transporters, leading to higher signal variability and less signal accuracy in affective disorders. From a Boltzmannian thermodynamic and energy-production perspective, the option of reversibility to a healthier time-to-action, reducing entropy is implied. We employed logic gates to show deviations from healthy level-wise communication and the reversed conditions through compensations implying the role of non-neural cells and the extracellular matrix in return to excitation-inhibition balance and accuracy in the time-to-action signaling.

## 1 INTRODUCTION

Timely goal-oriented behaviors are vital for survival, shaped by experience [1] and refined through human evolution [2]. The time-to-action for goal-oriented behavior has been traditionally measured by reaction time (ranging from 800-1000 ms for different healthy prototypes) [3]. In healthy individuals, timely goal-oriented behavior is affected by environmental and age-dependent events requiring quick or slow reactions according to a given situation and the individual’s age. Additionally, each environmental stimulus or given situation may require slower or quicker reaction rates, which may be considered adaptive, promoting, and healthy or maladaptive, suppressing and disease-related. Accordingly, the mechanisms underlying goal-oriented behavior have been shown in the past decade to be complex, consisting of several levels of polymorphic, molecular, neural and non-neural signaling [4] driven by parallel sequences [5].

Pathological deviations from the healthy range of the time-to-action scale are apparent when behavioral reactions show discrepant gaps between the environmental time requirements and the actual reactions of the individual. In affective disorders, propagation delays of time-to-action were found to be associated with parallel self-referential thought, evaluative judgment and social cognition, while increased severity of depressive symptoms was associated with less frequent occurrences of a hybrid brain network implicated in cognitive control and goal-oriented behavior [6].

Updated mapping results suggest that a deficit in the anterior connectivity between the Default Mode Network (DMN) and the Salience Network (SN) may underlie abnormal connectivity dominance of the DMN, while an insufficiency in the anterior DMN-posterior DMN neural crosstalk leads to an abnormal dominance of the SN. These deviations from healthy connectivity have been suggested as underlying excessive individual subjective focus on internal contents and reduced shifting from idea to action. Those imply prolonged time-to-action (slower reaction) on the one hand, or on the other hand accentuated subjective centering on external inputs leading to a quicker switch from idea to action, which implies reduced time-to-action (quicker reaction). These two behavioral prototypes represent central behavioral dimensions, which are still serving to date for the diagnosis of clinical states of affective disorders, such as states of depression and mania [7]. In this paper, we aim to show the complexity of the time-to-action in affective disorders and its parallel underlying sequences which require a broader understanding rather than the classification of slow compared to quick reactions. Moreover, the aim was to show that signal inaccuracy, as resulting from clock-genes-based interference at the molecular, neuronal and non-neuronal functionality levels, represents the maladaptive, disease-related time-to-action.

### 1.1 Theoretical Framework

**Fig. (1):**
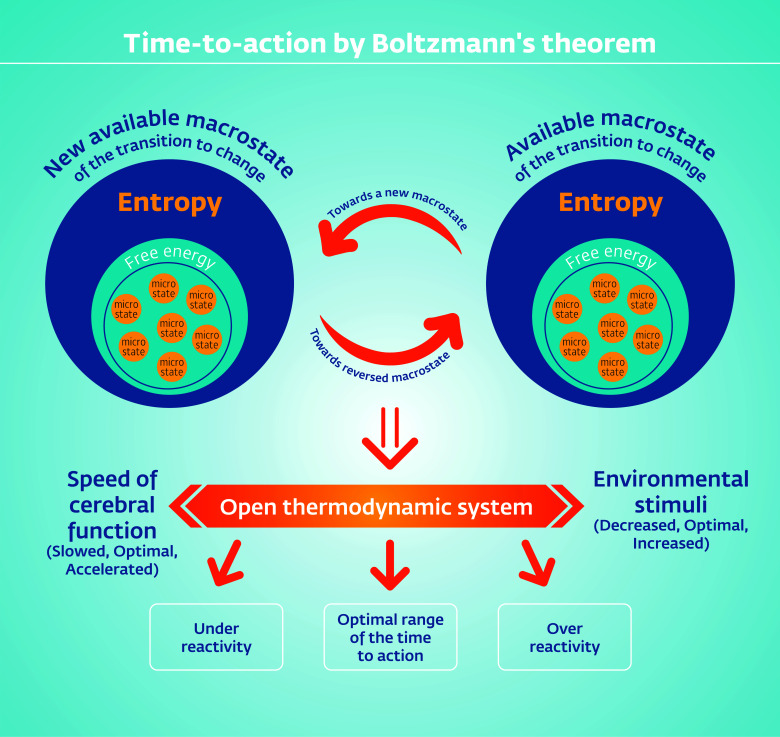
The healthy *vs*. psychopathological time-to-action as a result of the free energy-entropy axis at a given moment in a given environment and a given condition experience-shaped.

Our theoretical perspective adopts Boltzmann’s theorem [8-10] on a thermodynamic equilibrium where microstates accumulate to yield a given macrostate enabling reaction to an environmental challenge at a given time, age-dependent. The Boltzmann theorem also implies that macrostates occur on the thermodynamic axis of free-energy entropy in a reversible manner (Fig. **1**). Accordingly, the production of healthy goal-oriented behavior by a given macrostate reaches maximal equilibrium upon actualization of the goal-oriented behavior and, by that, ends this state while subjecting the thermodynamic system to a following macrostate towards the next accumulation of microstates and its resultant macrostate, which produces another behavioral output. This further implies that affective disorders are not fixed conditions of mental diseases but rather include a spectrum ranging from acute and reoccurring conditions to remission and regular functioning. These conditions changing at such a wide spectrum may enable viewing them as Boltzmann’s reversible macrostates, whose conditions depend on their timing at a given clinical status, while the aligned time-to-action differences of the same individual may differ between mental conditions. We argue that these pathological deviations from the healthy range of the time-to-action are environmentally dependent and age-appropriate and that these deviations occur on an axis ranging from healthy to psychopathological clocks rather than distinctly differentiating between “normal” and “abnormal” conditions. From a humanistic perspective, we suggest that these deviations are reversible when healthy macrostates become available, especially in responders to treatment.

Furthermore, the Boltzmann theorem implies that macrostates are generated in parallel at several levels of the available microstates within a thermodynamic system [8], such as the brain [11] and that they govern the time-to-action phases. According to the Boltzmann view, the time-to-action is the time of microstate generation up to a macrostate of maximal equilibrium followed by its exhaustion, leading the thermodynamic system towards the next available equilibrium (Fig. **1**).

In contrast, it has been found [12] that such excitation-inhibition balance is determined by XOR and XNOR logic gates representing regulated pathways in a multileveled parallel sequence of the cerebrum, suggesting that clock genes serve as “timers.” Unlike such balances in the healthy individual, the cerebral sequences in disease conditions employ other logic gates that dominantly control the resulting goal-oriented behavior by deviations of the time-to-action towards a lack of pruned regulated pathways. Those result in switches from the healthy free-energy-entropy matrix and imply a wider range of signal variability, including random signals and noise.

## 2 Connecting Boltzmann's Theorem with Studies of Affective Disorders

Unipolar and bipolar depression are not always as clear as the current categorical nosography (*e.g.*, DSM, ICD) seems to imply. In fact, the majority of patients with unipolar depression endorse features of bipolar depression, and a number of patients with unipolar depression will change their diagnosis to bipolar depression during their lifetime [13].

Boltzmann’s theorem implies that depression and mechanisms underlying bipolar depression stem from similar origins, including polymorphic, transcriptomic and molecular events. Therefore, they may be better described in a transdiagnostic manner of a continuum of unipolar and bipolar depression conditions, that is, the inclusion of both dysphoric and hypomanic clinical states in the same continuum. Here, we further argue that failure to maintain excitation-inhibition balance in both conditions, distorting this dual interplay to a pattern of just one type of molecular event, emerges from the inaccuracy of the level-wise clocks. Thus, the risk of running free continuously either on excitatory or inhibitory pathways alone depends on the number of communicating levels inverting the dual excitation-inhibition signaling balance.

Using Boltzmannian terms, in affective disorders, the following is exhibited: A failure to construct a level-wise macrostate caused by high entropy, which blocks the flexibility needed for the accurate changes between excitation and inhibition. This occurs along the collection of free energy parallel sequences production for a required goal-oriented behavior. Moreover, due to high degrees of entropy in affective disorders, the cerebrum’s efforts to collect microstates towards creating an environmentally adaptive macrostate are exhausted early in this process and returned to the starting point of collection. This leads to insufficient quantities of free energy for a goal-oriented behavior to be executed. Thus, this early exhaustion of partial collection of microstates prohibits a timely behavioral reaction, ending in repeated quick behavioral actions on the one hand or avoidance and behavioral activity blocking on the other. Unlike this pathology of the clocks, in the healthy organism, the macrostate, according to Boltzmann, is exhausted only following the execution of a behavioral action that comprises environmental appropriateness and the option of going forward to the next collection of microstates toward a new macrostate, leading to a new behavioral action. In this paper, we show that the failure to maintain XOR gates in the communication between the different neural levels pushes down this communication to primitive building blocks such as NOT gates. Those, unlike the dual inputs of XOR gates, have only one option for input and one option for output.

Following our line of thought arguing that the communication between the different levels of the neuron is facilitated by XOR logic gates [12] in the healthy cerebrum, the erroneous pathways are suggested here as decrementing to one input, not keeping the flexible pattern of XOR gates, endorsing the molecular events with either excitatory or inhibitory only command. Even if the NOT gate between the following two neural levels is corrected by another NOT gate (inverter), the outcome will shift from excitatory alone to inhibitory alone, thus lacking the XOR gate flexibility and deviating strongly from the excitation-inhibition balance toward one of the two signaling extremes. We term this underlying process “pathology of the neural clocks”.

We also argue that the potential rescue of the neuron from pathologies of its clocks involves the extracellular matrix and non-neuronal cells using two other primitive logic gates, OR and AND logic gates to restore the signaling of a XOR gate back to the neuron. This corresponds to Boltzmann's important formulation of macrostate reversibility which implies that the exhaustion of an available macrostate by execution of a behavioral action is afforded by reduced entropy and increased free energy for a healthier and trigger-addressing goal-oriented behavior.

Conditions of such erroneous communication, level-wise, can be applied to both uni- and bipolar depression on a continuum of a transdiagnostic axis, thus not viewing inhibitory conditions as representing only unipolar depression while not viewing excitatory conditions as representing only manic clinical statuses either. This suggests underlying mechanisms of pathologies of the clock, with shifts from preciseness in neural signaling to inaccuracy of underlying molecular events on the vector of time.

We note that this comprehensive focus on clocks in affective disorders is presented as a test case. Other psychopathologies included in the traditional diagnostic classification require separate attention and are characterized by the dominance of extreme excitatory (*e.g.*, Post-Traumatic-Stress-Disorder, Generalized Anxiety Disorder) or inhibitory (*e.g.*, Phobic types of disorders, Avoidant types of Personality Disorders) may be regarded as generated from erroneous neural level-wise communication and understood as pathologies of the neural clocks.

### 2.1 Aims

This manuscript aims to present the neuroscientific profiles of cerebral macrostates for a goal-oriented behavior, which are activated to comprise a complex set of timescales generated in a level-wise approach of parallel sequences. We argue that the pathway that determines the time-to-action in affective disorders is polymorphic, that it is affected by altered molecular events and that it eventually reaches a unique type of signal dominance and range of signal variability. The corresponding timescales do not comprise the time consumed for polarization alone but rather reflect the accumulated time from its onset at the polymorphic level and input at the molecular level up to producing the aligned subsequent signal variability, ending in its behavioral output. Thus, we aim to show that the summation of time starts from the onset of translational polymorphic activity through molecular and signaling feedforward and that a given goal-oriented behavior involves feedback progression, as we suggested earlier [12, 14]. However, we regard the level-wise events as parallel processes rather than a serial accumulation of periods for each process at each level. Hence, we imply that the healthy time-to-action is shorter than the simple summation of processes over time. This recognition of the time-to-action represents the creation of parallel sequences into an available macrostate, implying that psychopathological conditions determine level-wise deviations from the healthy range of cerebral timescales and inaccuracy of the time-to-action, including random signaling and noise. As these level-wise processes are generated at the polymorphic level, the timescales involved include individual differences, which can be validated by the particular progress of the disease process, as well as by the clinical conditions of reoccurrence and remission.

We thus argue that in affective disorders, the rhythms of time-to-action are comprised of complex mechanisms, reflected by ranges rather than by precise nano- or milli-seconds and that they thus cannot be judged simply by slow or fast reaction-time measures.

## 3 The polymorphic clocks

While the BDNF Val66Met polymorphism has been extensively investigated in affective disorders [15], emerging new data highlight the significant role of clock genes in these psychopathologies, especially regarding their role in the time-to-action that shows deviations from the healthy timescales of behavioral activity.

Cellular timing is controlled by over 18 clock genes [16]. These clock genes have been shown to be phase keepers in affective disorders by controlling the molecular and cellular input, contributing to the final output of signal variability [16, 17]. In affective disorders, these clock genes further display photic reactivity reflected in day-night phase advances, propagation delays and reduced amplitudes of circadian rhythms [18-20]. As we recently outlined [12], these clock genes are designed to function with on-off switches of free radical loads at shorter and longer timescales than their known 24-hour cyclicity [17, 21, 22].

Notably, the GGAC, AAAC and AGGA haplotypes of the clock gene CRY2 display excess in bipolar depression patients compared to controls, and their functioning involves rapid cycling and interaction of their protein products with each other [20]. Animal models further support the involvement of clock genes in depression and show that Clock and per2 are involved in the despair-like behavior of mice in the forced swim test [16]. From the prognosis perspective, the supportive role of clock genes has been shown in a mixed sample of patients living with unipolar or bipolar depression, where patients carrying polymorphisms of the per3 gene showed more novelty seeking and improved reactivity to treatment with SSRIs compared to non-responders without these polymorphisms [23]. Thus, beyond the resulting conclusion on the involvement of clock genes in affective disorders, and given that a mixture of genotypes related to them in these types of disease may reach almost 2 million combinations [24], these findings suggest that the preprogrammed role of clock genes as guardians of time is being switched, on the translational level to producing neural deviated signal transmission on a very wide range of outputs, including random signaling and noise [24]. This implies a lack of accuracy in the link between environmental triggers and aligned goal-oriented behavior. This magnitude of genotype mixtures could generate epistasis, further supporting our arguments that in affective disorders, deviations of signal variability from the healthy range of time-to-action are initiated at the transcriptomic and translational levels.

## 4 The RNA clocks

Small non-coding RNAs comprise the great majority of RNA sequences in all live organisms and provide an efficient level of regulation of messenger RiboNucleic Acid (mRNA) sequences. In particular, both microRNA (miRNA) and the recently re-discovered transfer RNA fragments (tRFs) may regulate gene expression by interacting with short nucleotide motifs on protein-coding mRNAs that carry regions complementary to their sequence, thus controlling the timing of cellular differentiation, growth and activity [25]. Feedback loops (positive and negative) connect Neurotrophins, tRFs and miRNA, all of which enable the modulation of various signaling pathways in the healthy individual, whereas their timely modulation is interfered with in psychopathology, including unipolar and bipolar depression [25].

The complex impact of non-coding RNA controllers on brain and body functioning is largely unresolved, although miRNA and tRFs rapidly acquire wide recognition as global controllers of regulatory processes, including their role in determining the time-to-action [12, 26, 27].

Notably, tRFs and miRNA may respond rapidly to stimulation by causing considerable changes in gene expression, which supports their role in regulating the timescales of behavioral actions and are suggested here as the RNA clocks of the time-to-action. This rapidly altered expression leads to altered quantities of target mRNAs, supporting our current concept of synchronization between RNA clocks governing the time-to-action of other clocks at other cerebral levels [28]. It was previously shown that tRFs interact with clock genes to determine the time-to-action for shorter and longer timescales than the 24-hour cyclicity and that their interaction is being calculated by the clock genes for dictating the cerebral free energy to actualize an action and its derived time until a behavioral output occurs [12].

One significant pathway of RNA modifications in psychopathological conditions, which interferes with the optimal excitation-inhibition balance, relates to the impact of regulatory RNAs on the cholinergic tone. The cholinergic pathway clearly involves clock gene activities, thus implicating a role of this pathway in determining the time-to-action in affective disorders. As the cholinergic tone and its regulation utilize inputs from the clock genes, especially in unipolar and bipolar depression conditions [29-31], any interference with this tone determines deviations from an optimal and healthy range of signal variability by affecting the parallel sequences of the clock functionality on other levels of neural activity for an adaptive behavioral action to be actualized on the vector of time.

Thus, we suggest that similarly to the impact of RNA alterations on the cholinergic pathway, parallel effects on additional pathways interfere with yet other molecular pathways, leading to deviations from the excitation-inhibition balance that is required for an optimal signal variability range observed in the healthy time-to-action. We further argue that these modifications significantly increase the signal variability range to the extent of random signaling resulting from the RNA clocks’ generation of multi-level noise signaling, interfering with the underlying signaling accuracy required for a healthy goal-oriented behavioral output.

## 5 The molecular clocks

Core molecular processes involving BDNF-NMDA receptor availability and the resulting neural changes in signal variability are affected in unipolar and bipolar depression. Under healthy conditions, crucial brain circuits display the sensitivity of the NMDA receptors’ excitatory function to fast inhibitory feedback [32] that regulates the time-to-action [12]. Healthy timescales are characterized by the coupling of signal rhythms and the modulation of gamma-band activities by theta and delta slower waves [33]. Specifically, the gamma band has been found to dominate rhythms in bipolar depression [34], while theta and delta bands appear to be dominant in depression [35]. The gamma band is modulated molecularly by the inhibitory neurotransmitter GABA, and it exerts dominant effects on the NMDA and BDNF receptors' activity through NMDA-BDNF interactions [36, 37], which in turn are generated by the GABA-NMDA molecular interaction [38]. Accordingly, NMDA receptor antagonists induce gamma band dysregulation of frequencies, including ongoing increased and reduced gamma oscillations [39]. Note that the effects on signaling are mostly based on Ca^2+^ ion channel gating and resulting postsynaptic activity [40, 41].

In pathological conditions, this dynamic may be dysregulated, leaving the neural cells under “takeover” by the inhibitory feedback or in excitotoxic states [32]. Accordingly, unipolar and bipolar depression appear to be characterized by excessive glutamate secretion overstimulating NMDA receptors [42] without appropriate regulation of reactivity to fast inhibitory feedback.

Treatments that block NMDA receptors or increase glutamate reuptake by astrocytes both decrease unipolar and bipolar depression’ psychopathology and increase neuroprotection and neuroplasticity [41, 43-47]. For example, lithium decreases glutamatergic transmission through NMDA receptors, thus protecting neurons from excitotoxicity [48]. BDNF is known to increase synaptic plasticity, and it is implicated, together with the NMDA receptor, in unipolar and bipolar depression [49]. Thus, the endpoint of reduced [36, 50-52] or over-increased [53] levels of BDNF is a crucial stage leading to a molecular cascade in affective disorders.

Several studies show that plasma/serum BDNF is decreased in unipolar and bipolar depression and that after successful treatment, BDNF retrieves normal levels [50, 51]. Similarly, reduced serum levels of BDNF were reported in both depressive and manic episodes and were normalized in euthymia to those of healthy subjects [36]. Accordingly, a large meta-analysis reported apparent peripheral BDNF dysfunction in affective disorders and mood dysregulation, especially in bipolar-diagnosed patients [37]. Furthermore, a meta-analysis of biomarkers in bipolar depression showed a negative correlation between blood levels of BDNF and depression severity score, supporting the role of BDNF in the depressive phase of bipolar depression [52]. This is compatible with reports of BDNF decline as bipolar depression advances, and factors that increase trauma and life stress are also associated with decreased BDNF levels [36]. Contrasting those reports, increased plasma BDNF levels were shown in patients with prolonged bipolar depression (over 20 years) [53]. These seemingly contradicting results support our argument for optimal ranges in healthy individuals and deviations from them in psychopathology.

We propose that the healthy timescales for a behavioral action to be taken are modulated by the interaction between receptors such as NMDA [38, 40, 54]. Molecular deviations from healthy timescales may alter the cortical axes responsible for the time-to-action [55]. As many crucial neuronal circuits in the brain are modulated by increased or decreased BDNF expression [56], the BDNF gene may initiate this cascade, leading to dysregulation of synaptic plasticity in affective disorders. Others suggested that alterations in the gamma band result from a GABA-NMDA imbalance that reveals molecularly induced inter-regional characteristics [34, 38, 57]. Interestingly, NMDA receptor diversity (hypo- and hyper-sensitivity to glutamate) acts as a complex mechanism modulating the excitation-inhibition balance [58, 59].

Thus, we suggest that a cascade of dysregulated BDNF secretion, which in turn affects NMDA receptors’ diverse reactivity on the inhibition-excitation axis, has consequences exemplified in deviations from a healthy range of signal variability. This cascade aggregates a disease-specific EEG wave in unipolar depression [35] and bipolar depression [34]. We argue that this process ultimately accumulates with time into a pathological molecular clock for the execution of inaccurate behavioral actions in affective disorders at a larger-than-needed variability of polarization-depolarization timescales.

## 6 The intracellular clocks

Intracellular clocks are affected primarily by the condition of alterations in mitochondrial function [60]. Alterations in mitochondrial function may, therefore, reflect differential changes in CNS cells’ metabolism and the influence that this has on patterned miRNA and Long noncoding RNA (LncRNA) expression, *i.e.*, an increase in mitochondrial oxidant production can alter patterned nuclear gene transcription *via* Reactive Oxygen Species (ROS)-dependent miRNAs [61], suggesting the multi-level parallel sequences of the time-to-action clocks.

In advancing the understanding of these parallel sequences of the clocks [12], we note that it is well-accepted that BDNF regulates the expression and traffic of NMDA receptors, which in turn are activated by the excitatory neurotransmitter glutamate’s impact on the mitochondria *via* downstream effects. Furthermore, in relation to the intracellular clocks, BDNF enhances Adenosine triphosphate (ATP) production *via* an increase in cell respiratory coupling, which provides the mitochondria-produced energy for the microstate to occur and accumulate. ATP is synthesized in the mitochondria, and both BDNF and NMDA rhythms were found to be responsive to on-off switches by clock gene expression through regulating Calcium ions' (Ca^2+^) quantity of entries to the cell [62]. Thus, the time-dependent functioning of mitochondrial energy production is crucial for determining the time-to-action of a goal-oriented behavior at a given moment for a given environmental goal.

Additionally, tRFs were found to protect the mitochondria by binding proteins, preventing apoptosis [63]. Unlike antioxidants such as melatonin, which is an oxygen scavenger, tRFs are cleaved in response to oxidative stress. This reduces their ability to protect the mitochondria [64]. Moreover, tRFs contribute to circadian crosstalk between mRNA metabolism and translation in healthy conditions, with propagation time enabling the accomplishment of mitochondrial energy production and respiratory phases of the cell in conditions of high metabolic demands [65]. Thus, under healthy involvement, tRFs prevent too speedy time-to-action, which results in a slower firing rate, reducing noise signaling of the cell without damaging the accuracy of the intracellular clocks.

The metabolic demands for energy production by the mitochondria result in the production of ROS and oxidative stress. Higher levels of oxidative stress have been found in affective disorders [66, 67], implying the dominance of excitotoxicity and NMDA receptor activation lacking regulated on-off switches by clock genes and their antioxidant products, such as melatonin.

Reduced melatonin levels in affective disorders are associated with elevated glucocorticoids [68], which, in turn, reduce the utilization of tryptophan, the precursor of the melatonin-serotonergic pathway [69-71]. This complexity allows a range of variable regulations of the melatonin-serotonergic pathway, including insults to the mitochondria of every cell type [72]. Such mitochondrial insults lack a significant feedback signal for regulated on-off switches of ROS by clock genes, Per1 CLOCK, Bmal1 and wider circadian genes [73].

Clock genes signaling is required to maintain ROS balance, and the production of their protein products interrupts mitochondrial insults, which threaten healthy transcriptomic patterns [22]. As such, variable clock gene expression is typical of affective disorders and can influence the timing of modulatory shifts between the levels of mitochondrial capacities to reach a healthy macrostate for a required goal-oriented behavior in cases of mitochondrial limited capacities that fail to produce the environmentally required energy for behavioral timely utilization.

Regarding the time-to-action, endogenous melatonin and melatonin supplements reduce activation in DMN-related regions [74], suggesting overactivation under conditions of mitochondrial excitotoxicity and longer times of polarization. Excitotoxicity in the form of flooding Ca^2+^ entries to the cell has been shown in both unipolar and bipolar depression [75]. This implies a shorter time-to-action in both unipolar and bipolar depression in a unique manner of increased noise on the signal-to-noise ratio, endangering cells’ fate and survival. Unlike the time-to-action in healthy individuals, in affective disorders, the time-to-action misses a link between intentions and specific goals, while the lack of clear goals implies the diffusion of actions [76]. In this manner, the impaired mitochondrial condition in affective disorders has a direct impact on alterations of the time-to-action since the energy-production status of this organelle prevents the creation of the environmentally required macrostate. Thus, the intracellular clocks in affective disorders are far from being accurate and likely to run freely, with a limited magnitude of macrostates, maximizing entropy.

## 7 The extracellular clocks

The Extracellular Matrix is the molecular composite outside the neuron, consisting of glycosaminoglycans, glycoproteins (laminin, fibronectin, tenascin, nidogen) and Fibrous proteins (collagens, elastin). Among its many functions, the extracellular matrix is responsible for reprogramming DNA insults and keeping the neuronal cells’ mitochondria healthy. Specifically, this matrix is known as the system supporting the return to molecular homeostasis and excitation-inhibition balance [77, 78], functions that imply the reversibility of macrostates in accordance with Boltzmann's theorem. As this matrix requires high volumes of energy for its function, it also leads to increased ROS, which, in turn, endangers the mitochondria. Clock genes serve as a source of control to keep the modulating function of this matrix in an optimal range, rescuing the organism from pathologies of the clocks. Findings show that damping of the clock in aging, for example [79], resulted in mitochondrial damage and prevention of reversibility of states [22, 80]. Thus, without the clock genes control, the cell remains at the mercy of oxidative stress, and its clocks deviate from the environmentally required time-to-action without assistance originating in the extracellular matrix.

The clock genes are designed to shift unstable molecules, including free radicals, into stable molecules by producing proteins that are electron transporters, such as the cascade leading to the glutathione transformation of unstable H2O2 into the stable water molecule [22]. Thus, deviations from the potentially positive role of the extracellular matrix may inhibit goal-oriented behavior or overexcite the firing of neural networks for rapid and repetitive goal-oriented behaviors in affective disorders. Furthermore, the condition of increased oxidative stress in both dysphoric and hypomanic conditions may cause situations of behavioral elimination or repetitive behaviors that lack clear goals on the vector of time. The advantage of the extracellular matrix is embedded in being able to return to balance and, by that, affecting the optimal range of firing rate within the entire neural network involved, including the clocks of the cell. As such, it serves, as known to date, as a target for the development of pharmacological means and non-pharmacological supplements for the treatment of unipolar and bipolar depression [81].

## 8 Non-neuronal clocks

A given microstate further combines the metabolism of astrocytes, microglia (and the microbiota that interact with them), along with the melatonin pathway for keeping these cells’ optimal functioning and actualizing their time-sensitive goal-oriented behaviors [82, 83]. As oligodendrocytes are responsible for the myelination of axons and the speed of signal transition, recent data show that defects in myelin production by oligodendrocytes lead to neural excitotoxicity resulting from inaccurate rapid signaling along axonal Ranvier Nodes [84]. Recent studies show that astrocytes and oligodendrocytes balance the accuracy of the firing of the neuron by local lipid metabolism of fatty acids within white and grey matter [85] along pruned pathways shaped by adaptive experience [12].

The summation of the metabolic demands of each of these cell types and the resulting free radicals at a given moment towards a given goal, experience-shaped, adds its share to the created macrostate by non-neuronal clocks. The reversibility of this summation (*i.e.*, the reversibility of an actualized macrostate) reduces excitotoxicity, leading to a more rehabilitated profile of microglia and gradual restoration of their resting states [86-88].

The control of clock genes to manage molecular demands of these cell types includes reduced amounts of free radicals that occur in every tissue [89, 90]. Furthermore, molecular analyses showed links between the central biological clock and the serotonergic system [91, 92], suggesting their involvement in affective disorders. Regarding reversibility, astrocytes are able to restore excitation-inhibition balance in the involved neural network [93], keeping a healthy range of time-to-action. Activated astrocytes are identified as either detrimental or protective, showing bidirectional transfer of mitochondria between astrocytes and neurons [94].

Following the debate over the last decade on the detrimental or promoting role of astrocytes, reduced numbers of astrocytes have been found in unipolar depression but not in bipolar depression [95, 96]. However, the conflicting results were found in studies that did not include the investigation of clock genes and their crosstalk with astrocytes or the balance between the detrimental and promoting astrocytes.

Thus, non-neuronal cells maintain a crosstalk with clock genes in every tissue [97]. This crosstalk is suggested here as the complex designed to maintain the summation of molecular events with time as “timers” of a goal-oriented behavior in healthy mammals by the required balance between detrimental and promoting astrocytes. The resulting time-to-action is dependent on this balance, while deviations from it imply the pathologies of non-neuronal clocks and inaccuracy in the time-to-action per environmental trigger in affective disorders.

## 9 The signaling clocks

EEG studies suggest that the underlying signaling of goal-oriented behavior in healthy *vs*. psychopathological conditions is characterized by differences in signal variability rather than by fixed times to action. A recent comprehensive review argues that individuals diagnosed with bipolar depression show less resting-task EEG variability compared to healthy controls [98]. Others showed increased slow wave power, which corresponds to greater signal variability in depressive conditions [99]. Accordingly, as shown in fMRI and EEG studies, an anticorrelation has been found between the midline regions' DMN and the lateral regions' Cognitive-Executive Network (CEN). Together, those networks characterize the balance between low (1-12 Hz) and high (30-180 Hz) EEG wave types, frequencies and their coupling in healthy conditions [33, 38].

Dysphoric conditions are characterized by higher signal variability of slower EEG waves, as shown by higher EEG power of the signal [99, 100]. In bipolar depression conditions, the gamma band (30-80 Hz) increases [101] at the expense of the delta and theta bands (delta: 1-3 Hz, theta: 4-7 Hz) [102]. This implies that in dysphoric conditions, low frequency delta and theta bands are apparent, while in hypomanic conditions, high frequency gamma bands increase and low frequencies decrease. The main cause for this imbalance in the stable anticorrelation is the shift towards infra-slow (fMRI: 0.0001-0.1 Hz) and slow frequencies (*e.g.*, [27, 103, 104]). Notably, these findings show that the main difference between these two types of signal variability in affective disorders is reflected in the degree of signal variability and wave coupling beyond the type of the signal. That is, larger signal variability of slow waves characterizes dysphoric states, while smaller signal variability of fast waves is apparent in hypomanic states. Importantly, wave coupling is lacking in both dysphoric and hypomanic conditions, showing that the complex inaccuracy in the time-to-action signaling clocks characterizes affective disorders as one disease entity rather than the simple comparisons of slower to faster disease-type reactions.

In support of this perspective, numerous studies indicate that antidepressants, including SSRIs and other medications, have been shown to reduce prefrontal theta wave cordance (a combination of absolute and relative (percent) power in a frequency band) of EEG spectra [105, 106]. Both mean theta and alpha (occipital) frequencies were slowed down after treatment with antidepressants, while the mean total frequency was accelerated at frontal sites and decreased at occipital sites [107]. Accordingly, diagnosed bipolar patients treated with Lithium showed downregulation of gamma band dominance, which decreased gamma amplitude by downregulating Ca^2+^ [108]. Lithium also increased the magnitude of oscillations in slow frequencies (delta and theta) in bipolar-diagnosed patients [34, 109].

As the responsivity of NMDA and BDNF to clock genes’ feedforward time regulatory signaling has been recently shown [62], we hypothesize that the fast molecular reaction of NMDA to inhibitory feedback in healthy conditions of over-excitatory glutamatergic input is the basis for the optimal range of signal variability which implies accuracy in the time-to-action. This input, in turn, is modulated by BDNF’s synaptic regulation and its following cascade of parallel interactive BDNF-GABA-NMDA sequences. Thus, we suggest that an optimal variability range of wave oscillations and wave coupling represent the accuracy of signaling clocks in healthy conditions. Those are based on neural and non-neural regulation of firing rate by molecular excitatory and aligned inhibitory feedback, employ available molecular and metabolic resources and are responsive to clock genes resolution of the resulting ROS.

## 10 Integration of findings (A): How do the level-wise clocks communicate and compensate for inaccuracy?

In this paper, it is demonstrated how the clocks deviate from the excitation-inhibition balance at each level. Additionally, the healthy pattern of level-wise communication has been detailed in our recent theoretical study [12], showing how XOR and XNOR logic gates govern communication between levels to maintain excitation-inhibition balance from the originating idea (the eliciting trigger) up to the actualization of a related goal-oriented behavior, termed as time-to-action.

**Fig. (2):**
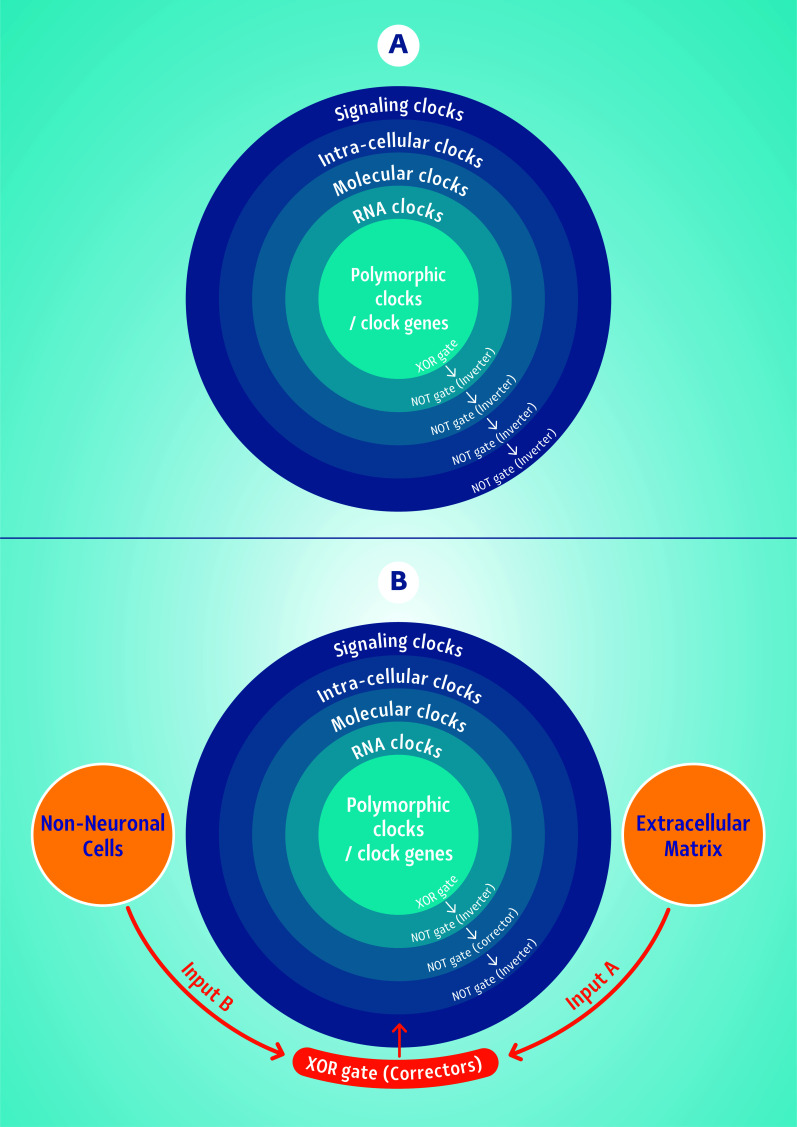
(A) The inaccurate clocks’ communication includes multi-level neuronal errors, where the healthy genetic XOR gate output signal is inverted multiply to NOT gates. This implies one continuous and erroneous signal of either excitation or inhibition rather than a flexible interchange between the two types of arousal levels. The outputs of every logic gate in the cascade along the multi-level function are shown as a function of logic gate levels in A and B (see also Table **1**). (B) The coordinated correction of the multi-level clocks in the neuron by the extracellular matrix (input A) and non-neural cells (input B) signaling involve a XOR gate function back to the neural cell (see also Table **2** and Fig. **3**).

It is proposed that the level-wise inaccuracy of the clocks in affective disorders is caused by an error, turning the healthy XOR gates into inverting NOT gates. Thus, if an excitatory signal has originated in the clock genes and related RNA levels, this signal is inverted to an inhibitory one. The opposite may occur with an original inhibitory signal, which is inverted into an excitatory one by the error of changing the healthy XOR gate into a NOT gate. Unlike XOR and XNOR gates, the invertor NOT gates have just one entry and, therefore, are not able to work with two inputs when either one of the entries is activated but not two at once. NOT gates invert any logic 1 output to 0 and any logic 0 output to 1 (Fig. **2** and Table **1**).

**Fig. (3):**
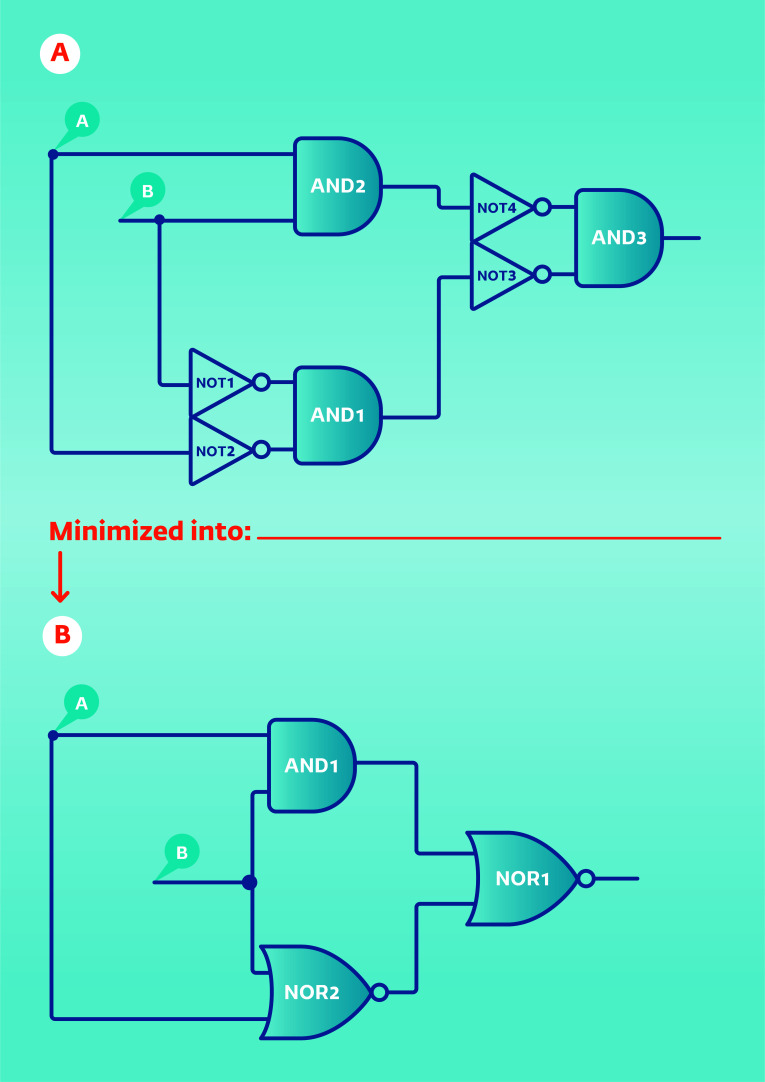
The logic complex for the return of the XOR gate function by primitive building blocks of AND and OR gates. (A) The logic version of the complex is shown in the upper part of the figure. (B) In the lower part, when A and B are both 0, the outputs of AND1 and NOR2 are 1 and 0, respectively. It can be verified that the output of the NOR1 gate behaves like an XOR gate in response to the logic levels of inputs A and B (see also Table **2**).

Notably, the brain has the resources to correct such errors, although these corrections retain their own limits. Such corrections are possible when the level next to the inverted level turns into another NOT gate and inverts the error to its correct origin, either excitation or inhibition (Fig. **2** and Table **1**). However, continuous inversions by NOT gates along the communication between the levels retain the risk of ending in an error signal. The potential assistance from non-neural and extracellular cells is available only if they coordinate as two types of inputs: A and B, which can work together as an XOR function by primitive building blocks such as AND and OR logic gates (Fig. **3** and Table **2**), meaning either accurate timely polarization or accurate timely depolarization along with all molecular events needed for either of these conditions. Maintaining NOT gates in the non-neural and extra-cellular cells will not add to the correction of the error in the neuron, will cause a collision between signals and will end in damping of the system.

A few molecular examples of such corrections of a pathological error signal and the erroneous shifting from XOR gates to NOT gates may be: (1) successful removal of the Mg^2+^ blockade, opening the Ca^2+^ entries to the cell with the timely return of the neuron to the resting state. (2) blockade of the BDNF cascade discussed above through regulation of the NMDA-BDNF-GABA secretion amounts and receptor availability by their related protein isoforms. (3) repeated selected reaction of the clock genes’ signaling to the extracellular and non-neural cells (with either 0 or 1 as outputs), enabling protection of the mitochondrial free energy production in the neuron. (4) clock genes’ activation of proteins that are electron transporters to reduce the burden of oxidative stress from the neuron. These types of cerebral compensations call for the next generation of pharmacotherapy.

## 11 Integration of findings (B): The level-wise complex in Boltzmannian terms

In Boltzmannian terms, in affective disorders, the order of the atoms and molecules involved is compromised (high entropy), blocking the probability of an adaptive macrostate to occur (maximal free energy), which would be behaviorally executed and then exhausted (maximal entropy) towards the next required behavior. Thus, the high entropy in the translational levels, such as the RNA level, implies the wide signal variability in other levels, including the rate of neural firing.

The larger-than-needed variability in polarization-depolarization timescales reflects excessive molecular cascades that are excitatory or inhibitory alone. These result in entropic pathways and decrements in the probability of an environmentally-required quantity of free energy molecular events. In Boltzmannian terms, they represent the repeated exhaustion of any collection of microstates created in pathological conditions. This contrasts with the healthy exhaustion of macrostates following the execution of an environmentally required goal-oriented behavior, providing the initiation of another microstate collection process within smooth parallel sequences.

This inaccuracy, in Boltzmannian terms, means that the collection of microstates is random, with limited resources of free energy for reaching a macrostate responding with a timely adaptive behavioral reaction to an environmental trigger due to the compromised conditions of the mitochondria. Thus, the wide variability range of pulses that equals 1 implies collisions in neural pathways rather than the creation of an appropriate macrostate for a timely goal-oriented behavior.

In Boltzmannian terms, the extracellular matrix and non-neuronal cells are sources of rescue of the neuron from inaccuracy of timescales. These are potential time-correctors of microstate collection processes progressing towards a macrostate, which can produce time- and goal-appropriate behavior.

Unlike wave decoupling found in affective disorders, in Boltzmannian terms, the normative wave coupling and its aligned molecular cascades represent the highest probability for a macrostate to occur in a timely manner. This reflects a required accuracy that may also be described as the time-appropriate ordering of particles involved in producing a healthy goal-oriented behavior. As such, the extracellular matrix, non-neuronal cells and their impact on timescales of wave coupling are suggested as future targets for pharmacotherapy.

The communication between the different levels and types of compensation through return and recreation of XOR gates can maintain the excitation-inhibition balance and the accuracy of molecular events, and those XOR gates are hence suggested as future pharmacological targets. Specifically, the protein isoforms to be identified as actively involved in the compensational process, especially those translated from clock genes, may be addressed by pharmacogenomics as maintaining the excitation-inhibition balance which parallels the accuracy of timescales.

## 12 DISCUSSION

To address the psychopathological clocks in affective disorders, we employed overarching data from recent findings. Those were based on Boltzmann’s theorem and post-modern scientific records of the entropy-preventing brain “timers” dictating the time-to-action according to available free-energy. We discussed the dependence of signal variability range, polarization and firing rate on the multi-level clocks’ time accuracy. This refers to the time-sensitive actualization of a behavioral action in the face of a given environmental stimulus, known in the scientific literature as goal-oriented behavior. We described when and how the multi-level clocks of the time-to-action work in parallel processes to accumulate microstates into an available and reversible macrostate. Finally, the deviations of the multi-level clocks from the healthy range of signal variability were observed on each level of clocks in affective disorders affecting level-wise signaling accuracy.

At the molecular level, we suggest that the diversity of the NMDA receptor subunits depends on the specific reactivity of polymorphic composites of the BDNF gene, such as the Val66Met variant and its interaction with clock genes and tRFs. This, in turn, determines timescales of goal-oriented phenotypes in affective disorders. Taken together, the time-to-action is reflected in altered gamma, theta and delta wave types, including their decoupling in affective disorders [34, 110], and is initiated at the RNA level, which differentially triggers molecular impacts on these waves and the range of their signal variability. In pathological conditions, the gamma band fails to be modulated by the molecular basis, generating slower theta and delta waves. This deviates both from healthy excitation-inhibition balance and from wave-coupling beyond the difference in the type of the signal band in depressive and manic clinical conditions [110]. Specifically, the healthy condition of wave coupling corresponds to the Boltzmann theorem of available macrostates for a given situation. In parallel, the pathological conditions of wave decoupling, such as those observed in affective disorders, correspond to the unavailability of environmentally-required macrostates and the eventual entropy dominance on the free-energy-entropy axis of cerebral thermodynamics, including mitochondrial risks. We showed that mitochondrial conditions reflect intracellular and extracellular imbalances in both neurons and non-neuronal cells. Moreover, they add to the inaccuracy of time-to-action clocks by increasing the dominance of entropy, impairing regular mitochondrial dependency on the feedforward of on-off switches by clock genes. Therefore, their transcriptomic and electron transporters protein isoforms are unable to accurately respond to mitochondrial feedback and fail to rescue their energy-production and respiratory phases from overloading ROS. Moreover, these events prevent microstate accumulation in reaction to a given environmental trigger at a given moment.

We further argue that the behavioral output in these psychopathological cases is the outcome of deviations from the healthy rate of signal variability dominance of signal type, accompanied by a lack of EEG wave coupling and significant differences in oscillations and their magnitude. These deviations suggest that the underlying clocks of pathological goal-oriented behavior are not simply the outcome of a slower or quicker neural activity [110] rather, those reflect altered and inaccurate time-to-action processes in affective states representing random polarization and excitotoxicity, eliciting either elimination of behavioral reactions or their repetition without a clear goal [33, 38, 99] (Fig. **1**).

Following our previous model on healthy level-wise communication by XOR gates [12], we herewith employed logic gates to show how pathological deviations and reversed conditions operate through logic compensations. Those, in turn, implicate non-neural cells and the extracellular matrix in the time-to-action signaling, enabling the return to excitation-inhibition balance and accuracy.

## 13 Further research and predictions

Our study proposes new modes for investigating the underlying parallel mechanisms of time-to-action in health and mental disorders. This could involve sampling blood for analyzing disease pleomorphic biomarkers at the genomic level, constituting RNA-sequencing-based mRNA and tRF profiles and hypothesizing molecular substrates concurrently with event-related electroencephalography (EEG) as in [111] across different environmental stimuli and at different ages. Using these tools to examine individuals diagnosed with affective disorders may yield comparative profiles of acute, chronic and remission clinical pictures. In comparison, the measures of those diagnosed as remitted could be compared to the timescales found in healthy controls. Additionally, the apparent sex-related differences between short RNA regulators of the cholinergic network in the brains of schizophrenic and bipolar patients [112] and the reported differences in the response patterns to therapeutics between men and women [113] indicate potential differences across sex types in a transdiagnostic manner. This, in turn, suggests potential sex-related differences in the psychopathological multi-leveled parallel sequences of timescales comprising the time-to-action.

## CONCLUSION

Psychopathological clocks, with emphasis on affective disorders, differ from healthy ones in the summation of the parallel sequences of the excitation-inhibition balance at each level and between levels. Thus, healthy *vs*. pathological timescales are not a simple phenotypic distinction between slow *vs*. quick time-to-action or an exact number of nano- or milliseconds of activity, nor do they represent a constant condition of disease. Rather, they encompass a range of signal variability that underlies the time-to-action in a manner that depends on the given genetic background and transcriptomic profiles of the individual, as well as on the environment, the timing of the situation for that given individual and his\her age, as well as on his\her experienced-shaped pruning.

Healthy *vs*. psychopathological timescales are determined by micro and macrostates of thermodynamic equilibrium, which correspond to excitation-inhibition balance between the RNA, molecular and signaling levels exhibited in the healthy temporal brain sequences of time-to-action. In this context, RNA regulators reflect the vast majority of transcribed sequences in the mammalian genome [114] and include both coding and non-coding sequences, short and long. The impact of those sequences affects the molecular cascades we have covered and regulates much of the networks that keep ongoing “timers” power to determine the time-to-action in health and disease; those span a BDNF cascade as well as the GABA-NMDA interactive sequences. Specifically, the joint impact of small non-coding RNAs and the neuronal networks involved supports our suggested concept that the psychopathological timescales encompass a complex of underlying mechanisms that function in parallel along the vector of time *via* different polymorphic, neuronal, non-neuronal and molecular levels of deviations from the excitation-inhibition balance, prolonging conditions of either excitotoxicity or inhibitory and signaling inaccuracy.

In healthy or remission conditions, the above regulatory levels create an optimal range of signal variability [12]. In affective disorders, there is a shift from the polymorphic and molecular excitation-inhibition balance affecting the dominance of connectivity between cortical structures towards a resultant increase in the slow wave signal variability and decline of global wave coupling under dysphoric conditions. In parallel, decreased signal variability of fast waves, along with lack of inclusive wave coupling, characterizes hypomanic conditions.

The efficacy of clock genes for on-off switches of oxidative stress by transcriptomic protein isoforms that function as electron transporters enables the transformation of unstable molecules such as ROS. Therefore, sequencing the timing of elevated and reduced secretion levels, including the timing of synthesis of oxygen scavengers such as melatonin, is highly impaired in affective disorders. The time-to-action in these pathological conditions may thus be misinterpreted by the measure of reaction time. The multi-level clocks, including extracellular, intracellular and non-neuronal clocks, affect the status of energy production and thermodynamic equilibrium by the mitochondria as well as the fate and survival of the cell while creating feedforward and feedback loops with clock genes. This, in turn, results in the programming and reprogramming of energy production for timely, healthy behavioral reactions. When on-off switches of clock genes fail to provide feedforward messages for an environmentally required mitochondrial energy production in response to a given trigger at a given moment, the multi-level clocks may run free and inaccurately at a large scale of signal variability.

According to the Boltzmann theorem, we argue that the differences in the complex underlying mechanisms of the time-to-action in affective disorders represent the unavailability of an environmentally-required macrostate (Fig. **1**). Accordingly, these are the consequences of the shift towards more entropy on the free energy-entropy axis and the lack of thermodynamic equilibrium to produce the goal-oriented behavior within an optimal range of signal variability, thus preventing the availability of the next macrostates and their reversibility. This excludes responders to treatment who serve, in turn, as the best evidence for Boltzmannian reversibility of macrostates. The modeling of erroneous pathological communication between levels and ways for compensation for those deviations from the healthy excitation-inhibition balance on the vector of time are shown using logic gates. We add to the Boltzmannian theorem the proposition that the reversibility of macrostates implies the return of accuracy to the time-to-action.

## Figures and Tables

**Table 1:** Shows the NOT gates inverters into states of either O (inhibition) or 1 (excitation) only.

A	B	XOR1	NOT1	NOT2	NOT3	NOT4
0	0	0	1	0	1	0
0	1	1	0	1	0	1
1	0	1	0	1	0	1
1	1	0	1	0	1	0

**Table 2:** Shows the return of the flexible excitation-inhibition balance implied in the XOR gate function as recreated by primitive building blocks of AND and OR logic gates.

A	B	AND1	NOR2	NOR1
0	0	0	1	0
0	1	0	0	1
1	0	0	0	1
1	1	1	0	0

## LIST OF ABBREVIATIONS

ATP: Adenosine Triphosphate
CEN: Cognitive-executive Network
DMN: Default Mode Network
EEG: Electroencephalography
LncRNA: Noncoding RNA
miRNA: microRNA
ROS: Reactive Oxygen Species
SN: Salience Network
